# Disparities in Women With Endometriosis Regarding Access to Care, Diagnosis, Treatment, and Management in the United States: A Scoping Review

**DOI:** 10.7759/cureus.38765

**Published:** 2023-05-09

**Authors:** Shannon Westwood, Mackenzie Fannin, Fadumo Ali, Justice Thigpen, Rachel Tatro, Amanda Hernandez, Cadynce Peltzer, Mariah Hildebrand, Alexnys Fernandez-Pacheco, Jonathan R Raymond-Lezman, Robin J Jacobs

**Affiliations:** 1 Dr. Kiran C. Patel College of Osteopathic Medicine, Nova Southeastern University, Fort Lauderdale, USA

**Keywords:** general gynecology, gynecological disorder, racial and ethnic disparities, surgical complication, women’s health

## Abstract

Endometriosis is a benign gynecological condition that elicits chronic pain in 2-10% of reproductive-age women in the United States and exists in approximately 50% of women with infertility. It creates complications such as hemorrhage and uterine rupture. Historically, the gynecologic symptoms of endometriosis have been associated with economic strain and inferior quality of life. It is suspected that endometriosis diagnosis and treatment are affected by health disparities throughout gynecological care. The goal of this review was to collate and report the current evidence on potential healthcare disparities related to endometriosis diagnosis, treatment, and care across race, ethnicity, and socioeconomic status. This scoping review followed the Preferred Reporting Items for Systematic Reviews and Meta-Analyses (PRISMA) guidelines and searched the Excerpta Medica Database (EMBASE), Medline Ovid, Cumulated Index to Nursing and Allied Health Literature (CINAHL), Web of Science, and PsycInfo databases for relevant articles on the topic. Eligibility was established *a priori* to include articles written in English, published between 2015-2022, and reported on cohort, cross-sectional, or experimental studies conducted in the United States. Initially, 328 articles were found, and after screening and quality assessment, four articles were retained for the final review. Results indicated that White women had higher rates of minimally invasive procedures versus open abdominal surgeries than non-White women. White women also had fewer surgical complications compared to all other races and ethnicities. Black women had higher rates of perioperative complications, higher mortality, and spent more time in the perioperative stage than any other race or ethnicity. In the management of endometriosis, the limited research available showed that all non-White women encountered an increased risk of perioperative and postoperative complications compared to White women. More research is needed to explore diagnostic and treatment disparities beyond surgical management, socioeconomic barriers, and improved representation of racial and ethnic minority women.

## Introduction and background

Endometriosis is a chronic inflammatory disease that globally affects up to 10% of reproductive-aged women [1]. Defined as extra-uterine lesions composed of endometrial glands and stroma, the estrogen-dependent disease process is often associated with chronic pelvic pain, infertility, pregnancy complications, and a decreased quality of life [2,3,4,5,6]. Due to its dependence on ovarian cyclicity, endometriosis commonly affects women between menarche and menopause [2,7]. Although clinically benign, the harmful impacts on quality of life are significant. Pain and associated dysfunctions are detrimental to patients’ economic status and professional, educational, social, and family lives, proving endometriosis to be a significant medical, social, and economic issue [8,9].

While a clinical history of dyspareunia and dysmenorrhea may raise suspicions of endometriosis, the non-specific nature of these symptoms is mimicked by several other potential etiologies. Additionally, endometriosis lacks pathognomonic features and biomarkers specific to the disease process [10]. Instead, diagnosis relies upon surgical visualization and histological confirmation of extra-uterine lesions consisting of endometrial glands and stroma [11]. However, the presence of these lesions cannot exclude other potential etiologies for the patient’s symptoms, nor can the absence of lesions prevent endometriosis. The lack of sensitive, specific, non-invasive diagnostic methods contributes to a delay in the diagnosis of endometriosis that can last between four and eleven years, contributing to unnecessary, prolonged suffering and decreased quality of life in patients [10,12].

Endometriosis is a complex disease with a multifactorial etiology, and several theories surrounding its origin exist. The most widely accepted theory of what causes endometriosis is retrograde menstruation, where endometrial cells flow backward through the fallopian tubes. The cells move into the pelvic cavity, where they can insert and grow. Cellular dysplasia of extra-uterine cells into endometrium-like cells has also been proposed, as has the theory that stem cells can give rise to the disease [13,14]. Other etiological factors may include altered or impaired immunity, complex hormonal influences and fluctuations, genetics, and environmental contaminants [15,16].

On average, globally, it takes seven years to diagnose endometriosis after the onset of symptoms, causing delays in diagnosis and subsequent treatment [17]. More than four million women of reproductive age have an endometriosis diagnosis in the United States; six out of ten cases go undiagnosed [12]. Historically, endometriosis was commonly known to affect White women while being seen as a “rare condition” for other races, leading to misdiagnoses for chronic pelvic pain in non-Whites [18]. This review explored the literature on endometriosis diagnosis, management, and treatment as it pertains to health inequities among women. 

## Review

Materials and methods

This scoping review followed Preferred Reporting Items for Systematic Reviews and Meta-Analyses (PRISMA) guidelines. Eligibility was established a priori to include articles written in English, published between 2015-2022, and reported on cohort, cross-sectional, or experimental studies conducted in the United States. Initially, 328 articles were found, and after screening four were retained for the final review. 

Search Strategy 

This scoping review searched Excerpta Medica Database (EMBASE), Medline Ovid, Cumulated Index to Nursing and Allied Health Literature (CINAHL), Web of Science, and PsycInfo with completed searches on September 26, 2022. The search strategy was created in EMBASE by the first author and reviewed by an expert librarian. The goal of the search was to uncover what evidence is available on disparities in endometriosis care in the United States. The EMBASE search strategy was adapted for Medline Ovid, CINAHL, Web of Science, and PsycInfo. The search terms used were “endometriosis,” “endometrioma,” “social inequality,” “health care disparity,” “health care access,” “economic,” “income,” “gender,” “sexual, bias,” “disparities,” “minority,” and “poverty.” Multiple combinations of search terms were used to obtain maximum results. The citations and bibliography sections of the included articles were reviewed; no articles were found by other means (e.g., hand searching) that met the search criteria.

The four articles that met inclusion criteria were evaluated for method quality and the risk of bias using the Joanna Briggs Institute (JBI) critical appraisal tools. All articles were evaluated using the JBI critical appraisal checklist for cohort studies. All four articles were found to have a minimal risk of bias, with scores above 70%. No articles were excluded based on the JBI checklists.

**Figure 1:**
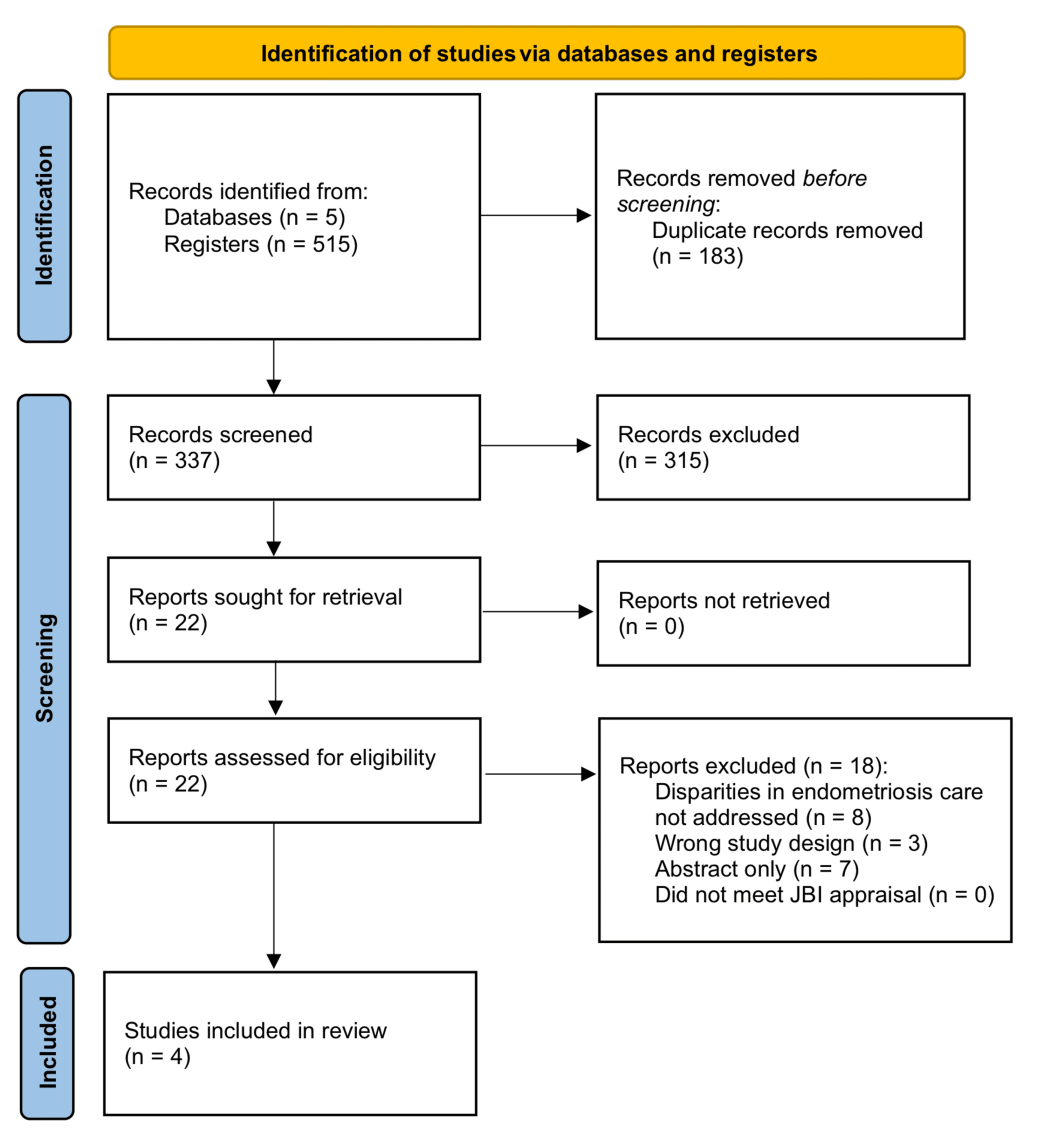
PRISMA Flow Diagram of the Selection and Screening Process PRISMA: Preferred Reporting Items for Systematic Reviews and Meta-Analyses

Figure 1 portrays the selection and screening process.

Results

A total of four articles were retained for this review. All identified articles were retrospective cohort studies that addressed disparities in the surgical management and outcomes of women with endometriosis. The two main concepts that appeared were ethnic and racial inequalities in 1) surgical treatment and 2) surgical complications. Three articles demonstrated that White women are more likely to have minimally invasive surgeries versus open abdominal surgeries for endometriosis diagnosis or treatment compared to other racial and ethnic groups [19-22]. All four articles found that White patients had lower rates of surgical complications compared to other racial groups [19-22]. Each article included different racial and ethnic categories with the most limited racial consideration only evaluating Black and White patients [19], while the most inclusive study used American Indian or Alaska Native, Asian, Black or African American, Hispanic, Native Hawaiian or Pacific Islander, White, and unknown race to categorize patients [22].

*Disparities in Endometriosis Surgical Treatments* 

Hysterectomies can be used in endometriosis treatment and diagnosis, and there are many variations of this procedure, from open abdominal laparoscopy to video-assisted laparoscopic surgery. Although minimally invasive hysterectomies shorten recovery time, decrease the length of hospital admissions, and decrease the risk of complications, there is still a disproportionate use of minimally invasive hysterectomies in White patients compared to minority racial and ethnic groups [19]. An article that specifically evaluated Black and White patients found that Black patients experienced a significantly higher rate of open hysterectomies compared to White women [19]. Another study investigating endometriosis treatment used a broader range of race and ethnicity categories and found that the highest rates of open hysterectomies occurred in Asian patients, followed by Black and Hispanic patients, while White patients again had the lowest rates of open abdominal hysterectomies [21]. A third study found comparable results showing that all racial and ethnic groups, except American Indian or Alaskan Natives, were more likely to have an open abdominal hysterectomy instead of a laparoscopic hysterectomy for endometriosis compared to White women [22].

The study with the broadest race and ethnicity categories explored the outcomes of surgical procedures for endometriosis beyond hysterectomies [22]. They evaluated eight surgical procedures to treat and diagnose endometriosis, including hysterectomies, and determined the rates of minimally invasive routes versus laparotomy. When the eight alternative endometriosis surgeries were considered, all racial and ethnic groups, excluding Asians, were more likely than White patients to have an open surgery instead of the minimally invasive alternative. When hysterectomies were excluded from the surgical types, Hispanic, Black, Asian, and unknown race/ethnicity patients were less likely to receive the minimally invasive surgical alternative. Black and Hispanic women had the highest rates of oophorectomy while overall hysterectomy rates were highest in White and Native American patients [22].

Disparities in Endometriosis Surgical Complications

The disproportionate use of open hysterectomies in non-White patients is accompanied by an increased risk of both perioperative and postoperative surgical complications [19-22]. In the studies evaluating the prevalence of major and minor postoperative complications, the Clavien-Dindo classification was used to distinguish major from minor complications [19,21]. The Clavien-Dindo classification ranks surgical complication severity based on the therapy type needed to fix the complication. The scale consists of grades I, II, IIIa, IIIb, IVa, IVb, and V. Grade 3 and higher are considered major complications and include cardiopulmonary arrest, myocardial infarction, venous thromboembolism, cerebrovascular accidents, pneumonia, renal failure, deep or organ surgical site infection, sepsis, unplanned reoperations, and death. Grade 2 and lower were considered minor complications and included blood transfusions, urinary tract infections, and superficial wound infections [19,21].

After accounting for confounding variables such as comorbidities and surgical approach, it was found that Black women had a significantly increased likelihood of experiencing major and minor postoperative complications than their White counterparts [19,21]. An article specifically evaluating the prevalence of lower urinary tract infections post-hysterectomy found that Black patients had higher rates of this complication than White patients [20]. This study also found that Black women had more extended hospital stays, unplanned readmissions, reoperations, and other adverse events in the 30 days postoperation. A study including Hispanic, Black, Native Hawaiian or Pacific Islander, and American Indian or Alaska Native women patients found that non-White patients were more likely to experience similar complications to those above postoperatively than did White patients, with Black patients having the most postoperative complications [22].

In addition to the increased risk of postoperative complications, Black patients had higher rates of perioperative complications compared to other racial groups [20,22]. Black women experienced higher rates of morbidity and mortality during surgery than their White, Hispanic, Native Hawaiian or Pacific Islander, American Indian, or Alaska Native counterparts [22]. Black women also had longer perioperative times than White women after accounting for confounding variables. Table 1 displays the characteristics of the articles in the review.

**Table 1:** Summary of findings of the four selected studies

Reference	Study Design	Study Objective	Race / Ethnicity Considerations	Major Findings	Limitations
Alexander, 2019 [19]	Retrospective Cohort Study	Evaluate women getting hysterectomies for benign gynecologic conditions for associations of race, surgical route of hysterectomy, and postoperative complications.	Black, White.	Black women were most likely to experience major and minor complications and surgery via an open hysterectomy compared to White women.	Not representative of all racial groups.
Bretschneider, 2019 [20]	Retrospective Cohort Study	Evaluate postoperative hysterectomy patients for the incidence of lower urinary tract complications and other factors during surgery for benign gynecologic indications.	Black, Other (Alaska Native, American Indian, Asian, Native Hawaiian, Pacific Islander), Unknown, White.	Increased risk of urinary tract complications was associated with Black race, endometriosis, and prior abdominal surgery after hysterectomy for benign gynecologic conditions.	Not representative of all racial groups. Multiple groups combined into “Other”
Movilla, 2022 [21]	Retrospective Cohort Study	Evaluate if complications from hysterectomy surgery for the treatment of endometriosis vary between different races and ethnicities.	Asian, Black, Hispanic, White	White patients were most likely to have laparoscopic hysterectomies, and Asian patients were least likely. Black patients were most likely to have major complications.	Possible sampling bias. Excluded “unknown/not reported” race and ethnicity women.
Orlando, 2022 [22]	Retrospective Cohort Study	Investigate disparities in surgical interventions or surgical complications for endometriosis related surgeries among different races and ethnicities.	American Indian or Alaska Native, Asian, Black or African American, Hispanic, Native Hawaiian or Pacific Islander, Unknown race, White	White women were most likely to have minimally invasive hysterectomies and fewer perioperative complications than minority women.	Could not account for possible confounding variables that were not included in the database used to obtain patient data.

Discussion 

This scoping review was conducted to evaluate the current literature available that addressed disparities associated with endometriosis diagnosis and treatment. A search of five scientific databases yielded four articles that met the inclusion criteria. All four articles showed racial and ethnic disparities in the care of women with endometriosis.

Two common themes across the studies suggested that White women were more likely to receive minimally invasive surgical options than ethnic and racial minorities [19,20,22] and White women were least likely to have surgical complications from their endometriosis-related surgeries than their racial and ethnic minority counterparts [19-22]. The disproportionate use of open surgical intervention in minority groups is significant because the minimally invasive options decrease the morbidity of the surgery compared to the open surgical options [23]. The lower rates of minimally invasive procedures among minorities likely predispose them to more surgical complications and worse outcomes. The expected higher rates of complications associated with decreased usage of the minimally invasive options were shown in all four articles. Minority women are more likely to have minor and major surgical complications, including urinary tract infections, urinary tract injuries, pulmonary embolism, and the need for a reoperation [19-22].

Previously published research supported the existence of racial and ethnic disparities throughout healthcare, despite controlling for socioeconomic factors and disease characterization [24]. This reinforces the current finding that non-White women receive inadequate endometriosis treatment in comparison to their White counterparts.

Limitations 

Included Studies

A limitation of the included studies is that they are all cohort studies that used the American College of Surgeons National Surgical Quality Improvement Program (NSQIP) database. The cohort studies depended on data collection from a database that might have a bias in data collection and other factors that cannot be controlled, including the location of the facility where the procedure was performed or the experience level of the surgeon [19]. The data evaluated for this study are from the hospitals that report their outcomes to the NSQIP. Since all articles used the NSQIP database to obtain their cohort data, the information might not be representative of the United States as a whole [19-22]. Another limitation is that the articles did not address the socioeconomic characteristics of the patients and only addressed surgical diagnosis and treatment of endometriosis [19-22]. Due to this lack of socioeconomic evaluation of the patients, these studies failed to address if the financial status, presence, absence, or type of health insurance held by the patient had an impact on aspects of health outcomes, such as when the patient sought medical evaluation if financial status prevented a surgery that was otherwise indicated or the type of surgery selected for each patient. The available studies focused on the surgical aspects of endometriosis, despite the search strategy including all diagnostic and treatment options. The absence of studies that discuss disparities in non-invasive medical management and outcomes of endometriosis care highlights the need for further investigation into the disparities in endometrial management.

Review Process

The review process yielded four studies that fit the inclusion criteria, which is a small number when considering that up to 10% of females have endometriosis, a chronic condition that is associated with chronic pain, decreased quality of life, and infertility [2]. The search was restricted to studies published in 2015 or later and data collected within the United States, which excluded older studies and some completed in other countries. The limitation on publication date excluded other relevant articles. Further, while this study included a small number of articles, the search strategy through five databases was robust, verified by an expert librarian, and conducted based on PRISMA guidelines. The articles were evaluated by multiple authors to determine if they fit the inclusion criteria, and all included articles were evaluated using the JBI critical appraisal tools. The reference list of all four studies was manually searched without revealing any additional relevant articles. 

Implications for practice

While the research studies contained in this review did not determine the cause of the disparity in outcomes for endometriosis treatment, the findings suggest that disparities exist for non-White minority groups. Until further research and conclusions are made, healthcare professionals and physicians should strive to ensure they provide consistently high-quality and appropriate care to all women seeking endometriosis treatment, regardless of race or ethnicity. Each patient should be apprised of all the treatment options and encouraged to work with their physician to find the best treatment to limit the risks of complications and adverse outcomes.

Future research considerations

The consensus of the four articles is that racial and ethnic minority females experience more complications from endometrial-related surgeries and are less likely to receive the laparoscopic surgical option. However, the causes of these findings were not well addressed. Future research should be conducted that aims to address if there are racial or ethnic biases from physicians and healthcare systems toward minority patients. Furthermore, studies should evaluate if there are confounding variables, such as socioeconomic or cultural factors, that are affecting the outcomes. Understanding the basis of why disparities exist is necessary to implement changes to correct the imbalance.

Future studies should aim to be inclusive of the racial, ethnic, and socioeconomic diversity of females with endometriosis. Studies that included only White and Black or White, Black, and Hispanic patients were not inclusive of the United States population. Excluding minority groups limits the ability to understand, address, and improve disparities within healthcare. In addition to the inclusivity of race and ethnicity, the socioeconomic factors that affect endometriosis care need further investigation. Exploration of how a lack of access to healthcare and the ability to afford treatment impacts those with endometriosis is necessary to address the disparity and decrease the suffering from this chronic condition.

This review only found articles about short-term surgical diagnosis and management related to endometrial care. Since endometriosis is a chronic condition that impacts the quality of life of the affected women, further evaluation should be conducted to determine if disparities exist in the long-term medical management of endometriosis [2].

Finally, to implement lasting strategies to improve and assure sustainable healthcare equity, there needs to be a prioritization of and commitment to conducting extensive, representative, and comprehensive research on the factors impeding health equity. Among these factors are minority attitudes towards the healthcare system, barriers faced in accessing quality care, and specific factors influencing provider decisions about the route of care, including provider bias.

## Conclusions

Regarding the diagnosis and surgical management of endometriosis in the United States, the research summarized in this review suggests that Black women face a higher risk of perioperative and postoperative complications in comparison to White women. Although one study found that American Indian or Alaska Native, Asian, Hispanic, and Native Hawaiian or Pacific Islander patients also face an increased risk of perioperative and postoperative complications, these racial groups are often overlooked in the current body of research. The preventable burden of suboptimal care experienced by non-White women must be further explored, along with contributing systemic and socioeconomic factors (e.g., income, insurance, and access to care), to implement best practices to ensure healthcare equity for all patients. Economic, educational, occupational, geographic, and sociocultural disparities are both embedded within and partially responsible for the systemic failure to provide minority patients with exceptional care. Non-White women face an unnecessary and problematic burden of risk when it comes to receiving quality endometriosis care, despite controlling for confounding variables.
