# Correction: Disparities in Women With Endometriosis Regarding Access to Care, Diagnosis, Treatment, and Management in the United States: A Scoping Review

**DOI:** 10.7759/cureus.c272

**Published:** 2025-08-26

**Authors:** Shannon Westwood, Mackenzie Fannin, Fadumo Ali, Justice Thigpen, Rachel Tatro, Amanda Hernandez, Cadynce Peltzer, Mariah Hildebrand, Alexnys Fernandez-Pacheco, Jonathan R Raymond-Lezman, Robin J Jacobs

**Affiliations:** 1 Dr. Kiran C. Patel College of Osteopathic Medicine, Nova Southeastern University, Fort Lauderdale, USA

This article has been corrected to fix the following typos in the PRISMA diagram:

- "Records screened (n=**332**)" has been corrected to "Records screened (n=**337**)"
- "Records excluded (n=**310**)" has been corrected to "Records excluded (n=**315**)"

